# Supplementary figures and images for: Immunogenicity of High-Dose MVA-Based MERS Vaccine Candidate in Mice and Camels
Source: Vaccines (Basel). 2022 Aug 17;10(8):1330. doi: 10.3390/vaccines10081330 (PMC9413082; doi:10.3390/vaccines10081330)

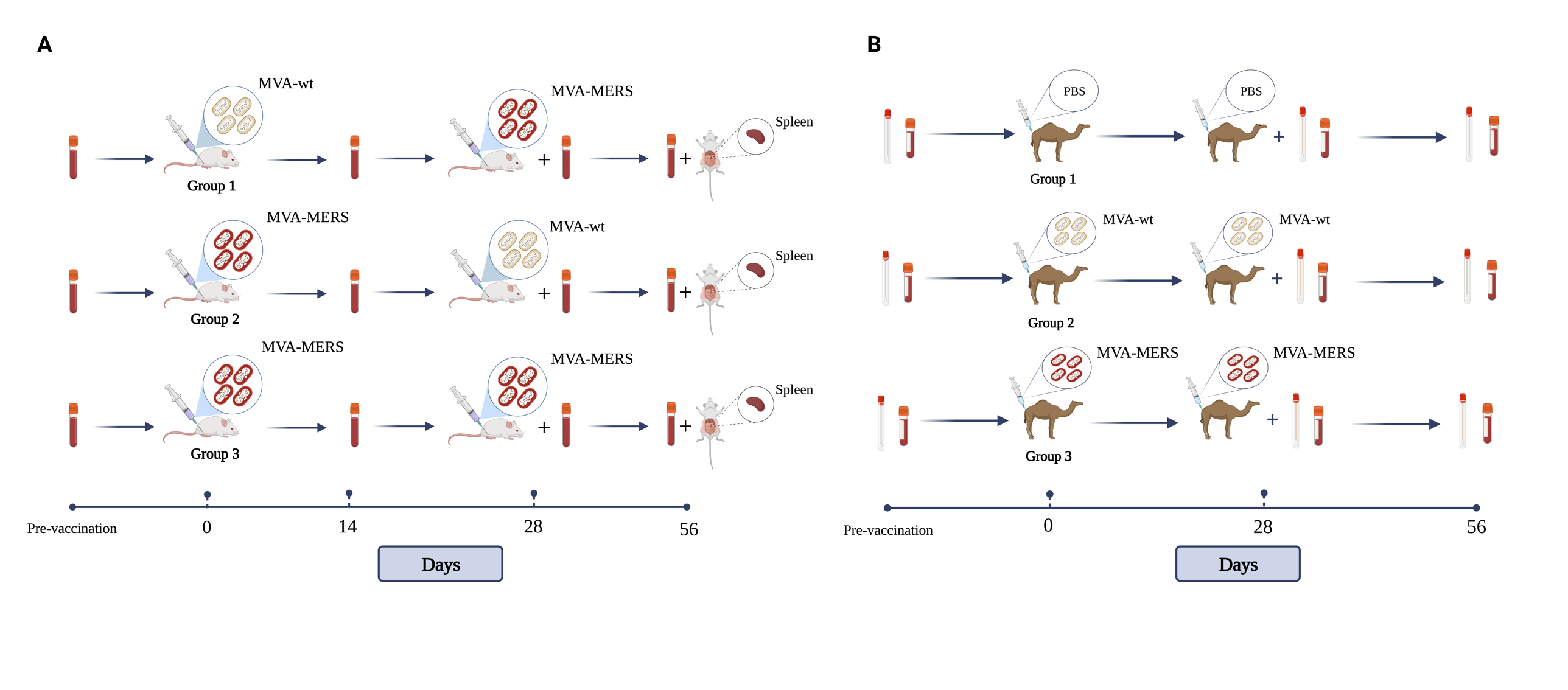

Supplement: Supplementary file 1 [file vaccines-10-01330-s001.zip › Figure S1.tiff]

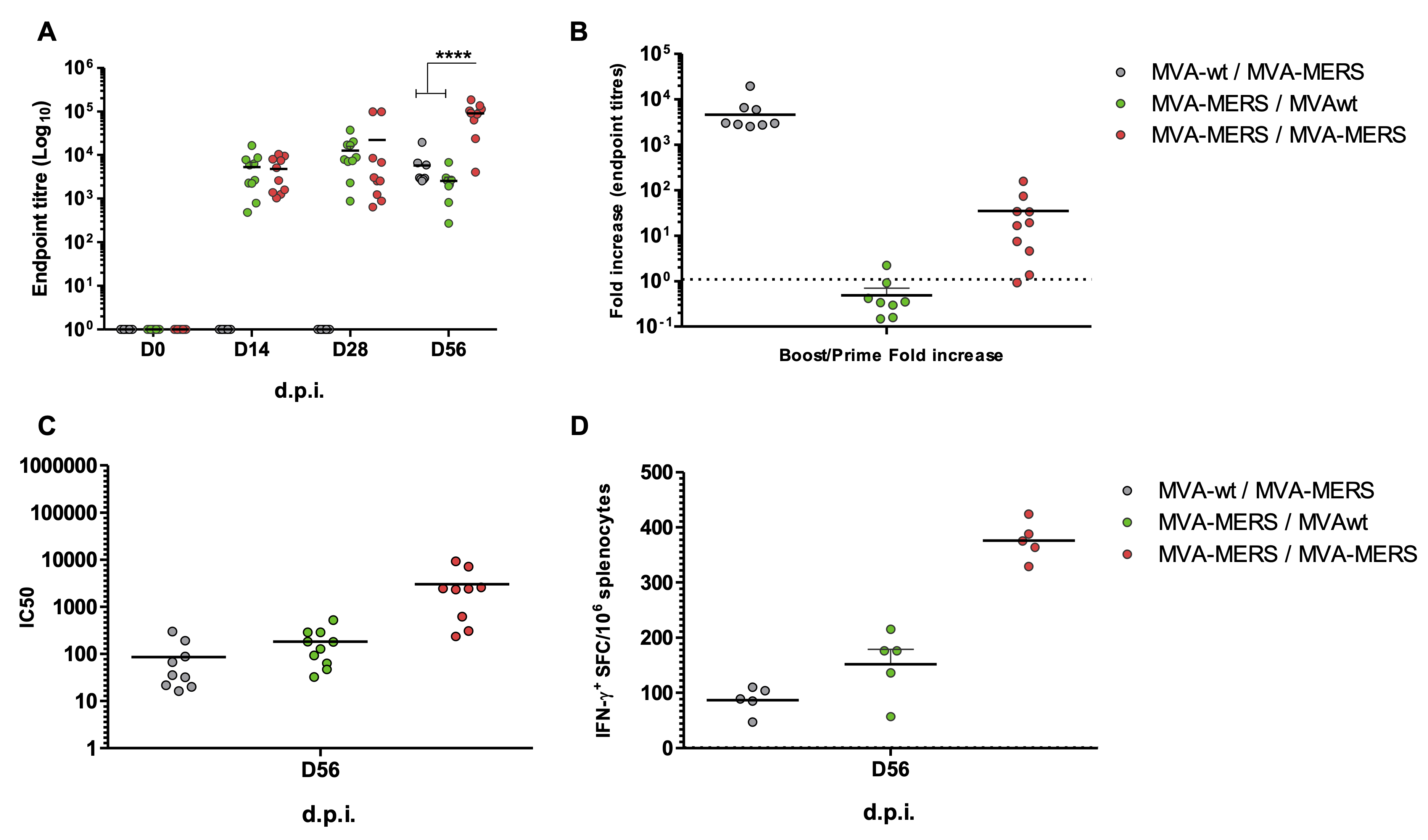

Supplement: Supplementary file 1 [file vaccines-10-01330-s001.zip › Figure S2.tiff]

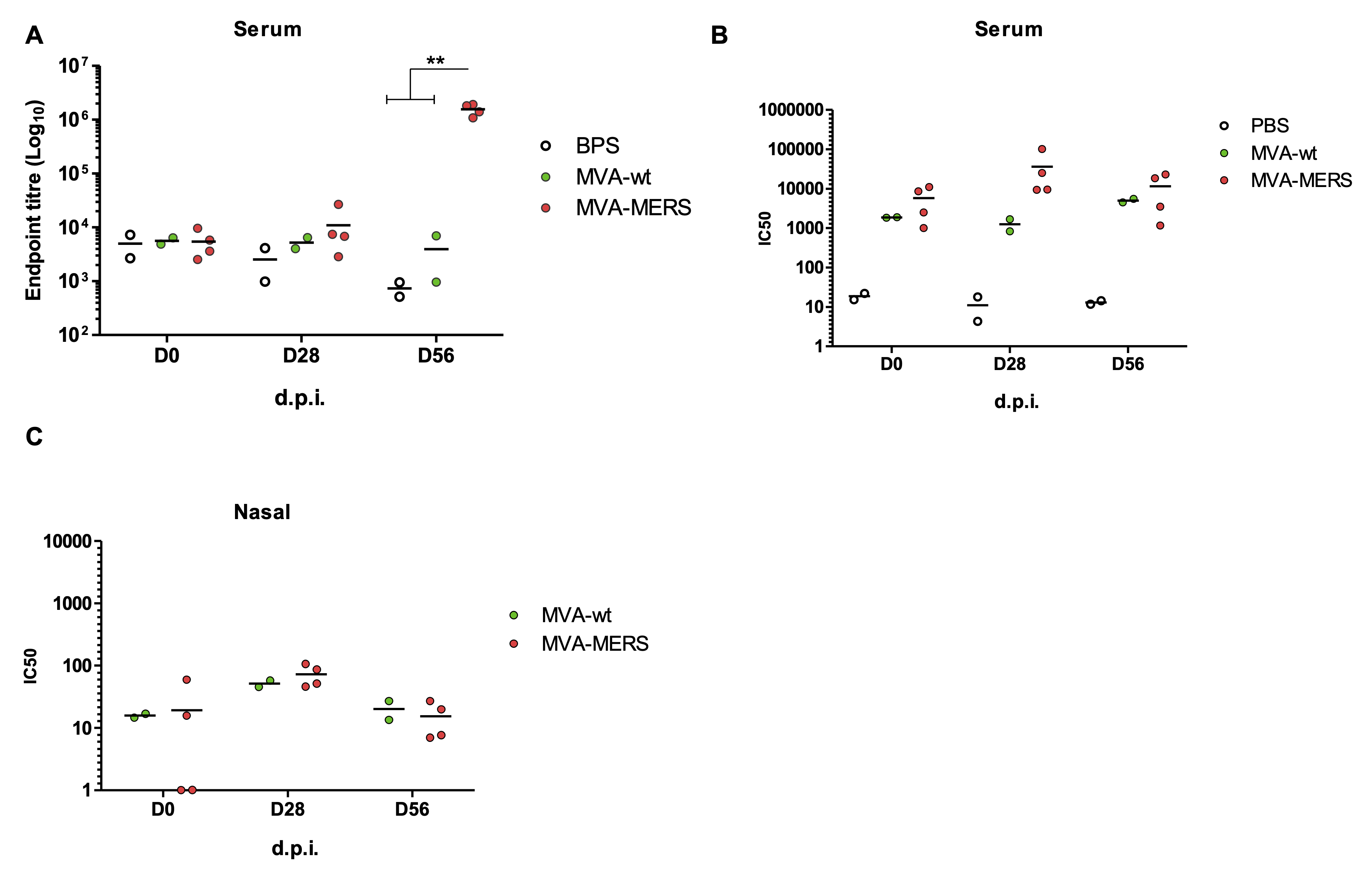

Supplement: Supplementary file 1 [file vaccines-10-01330-s001.zip › Figure S3.tiff]

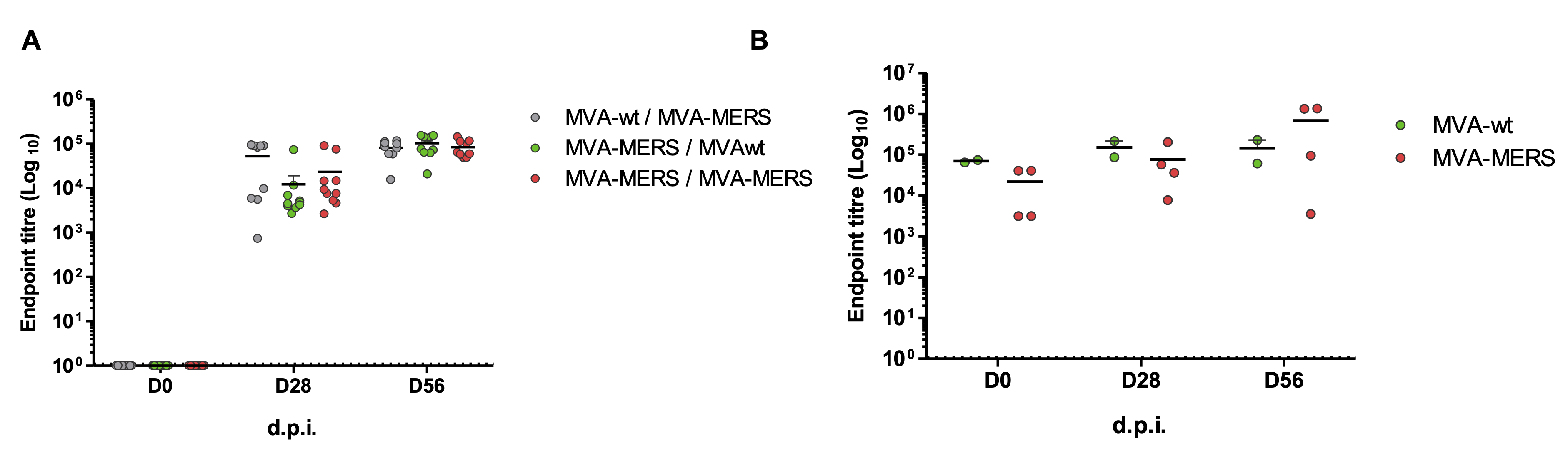

Supplement: Supplementary file 1 [file vaccines-10-01330-s001.zip › Figure S4.tiff]
